# Sequence and structural properties of circular RNAs in the brain of nurse and forager honeybees (*Apis mellifera*)

**DOI:** 10.1186/s12864-018-5402-6

**Published:** 2019-01-25

**Authors:** Clemens Thölken, Markus Thamm, Christoph Erbacher, Marcus Lechner

**Affiliations:** 1 Philipps-Universität Marburg, Institut für Pharmazeutische Chemie, Marbacher Weg 6, Marburg, 35032 Germany; 2 Julius-Maximilians-Universität Würzburg, Verhaltensphysiologie und Soziobiologie, Am Hubland, Würzburg, 97074 Germany

**Keywords:** circRNA, Circular transcriptome sequencing, Honeybee, Brain, Neuronal, Methylation, CpG, Alternative splicing, Behavioral plasticity

## Abstract

**Background:**

The honeybee (*Apis mellifera*) represents a model organism for social insects displaying behavioral plasticity. This is reflected by an age-dependent task allocation. The most protruding tasks are performed by young nurse bees and older forager bees that take care of the brood inside the hive and collect food from outside the hive, respectively. The molecular mechanism leading to the transition from nurse bees to foragers is currently under intense research. Circular RNAs, however, were not considered in this context so far. As of today, this group of non-coding RNAs was only known to exist in two other insects, *Drosophila melanogaster* and *Bombyx mori*. Here we complement the state of circular RNA research with the first characterization in a social insect.

**Results:**

We identified numerous circular RNAs in the brain of *A. mellifera* nurse bees and forager bees using RNA-Seq with exonuclease enrichment. Presence and circularity were verified for the most abundant representatives. Back-splicing in honeybee occurs further towards the end of transcripts and in transcripts with a high number of exons. The occurrence of circularized exons is correlated with length and CpG-content of their flanking introns. The latter coincides with increased DNA-methylation in the respective loci. For two prominent circular RNAs the abundance in worker bee brains was quantified in TaqMan assays. In line with previous findings of circular RNAs in *Drosophila*, circAm*rsmep2* accumulates with increasing age of the insect. In contrast, the levels of circAm*rad* appear age-independent and correlate with the bee’s task. Its parental gene is related to amnesia-resistant memory.

**Conclusions:**

We provide the first characterization of circRNAs in a social insect. Many of the RNAs identified here show homologies to circular RNAs found in *Drosophila* and *Bombyx*, indicating that circular RNAs are a common feature among insects. We find that exon circularization is correlated to DNA-methylation at the flanking introns. The levels of circAm*rad* suggest a task-dependent abundance that is decoupled from age. Moreover, a GO term analysis shows an enrichment of task-related functions. We conclude that circular RNAs could be relevant for task allocation in honeybee and should be investigated further in this context.

**Electronic supplementary material:**

The online version of this article (10.1186/s12864-018-5402-6) contains supplementary material, which is available to authorized users.

## Background

Honeybees (*Apis mellifera*) display a striking behavioral plasticity among their workers that is reflected in an age-dependent task allocation and thus represent a substantial model organism for phenotypic plasticity. Workers are able to execute varying specific behaviors in order to fulfill tasks that are essential for the viability of the colony, such as cleaning combs, feeding the larvae, guarding the nest entrance and foraging for food. To ensure economic efficiency and to prevent randomly performed tasks, the assignment of tasks has to be coordinated [1]. Task allocation is predominantly dependent on the age of the worker bees, but is also flexible and can be adjusted to colony needs [2–4]. In experimental single cohort colonies (SCCs) that are solely composed of young bees, some colony members initiate foraging precociously irrespective of their age [5]. Major differences in task-related behaviors exist between the typically younger nurse bees that feed the larvae inside the hive and the older foragers (≥18 days after emergence) that leave the hive to collect pollen, nectar and water [6–9]. This phenotypic plasticity is also reflected at the neuronal level. The overall brain volume is increased in forager bees compared to nurse bees [10, 11] especially in visually innervated brain structures [12, 13]. At synaptic levels, these changes involve for instance the density of synaptic complexes within mushroom body calyces caused by the growth of Kenyon cell dendrites and pruning of presynaptic boutons [14–16]. The regulation of these processes is poorly understood and seems to be highly complex. Various effectors are known which include the external environment, the colony state and internal stimuli such as (post-)transcriptional changes. Alterations in the expression ratio of hundreds of genes were detected, including some with synaptic functions [17–20]. Additionally, protein expression is affected as shown for instance for changes in neuropeptides [21], the membrane proteome [22] and the phosphoproteome [23] in the worker bee brain, but also in peripheral tissues such as the hypopharyngeal gland that produces royal jelly proteins [24]. Task- or age-related differences were also observed in the abundance of micro RNAs (miRNAs). Many of the identified miRNAs have a number of putative target genes that also exhibit functions in a neural context [25–27].

Circular RNAs (circRNAs) represent a class of RNA with considerable regulatory potential that was overlooked for decades and is currently under extensive research and discussion. An increasing number of studies show that circRNAs are abundant, differentially expressed and even have biological functions [28, 29]. In general, circRNAs arise from a back-splicing event. The 5’-end of a donor exon is joined to a 3’-end of an acceptor exon of the same molecule [30]. This results in a so-called back-spliced junction (BSJ) which can be observed as junction-spanning reads (JSR) when mapping RNA-Seq data to a genome using a mapper that supports split reads. The abundance of circRNAs typically varies between tissues and is sometimes uncorrelated to the host mRNAs [31]. This may indicate a specific function of circRNAs but might as well reflect distinct decay rates for linear compared to circular transcripts which lack accessible ends. Studies point out that circRNAs may act as regulators of alternative splicing [32] or could feature miRNA sponges [28, 33].

Besides human and mice, the presence of circRNAs was verified and studied extensively in the fruit fly *Drosophila melanogaster* and recently in the silkworm *Bombyx mori* but no other insect so far [28, 30, 31, 34]. Important findings are the presence of numerous miRNA seed matches in line with a putative miRNA sponge function as well as the fact that circRNAs mainly derive from neural genes and accumulate in neural tissues in an age-depended manner [32, 35, 36]. Following up on these findings, circular RNAs may contribute to regulating the age-related transition from nurse bees to foragers at the molecular level.

## Results

### Identification of circRNAs in the brain of honeybees

**Fig. 1:**
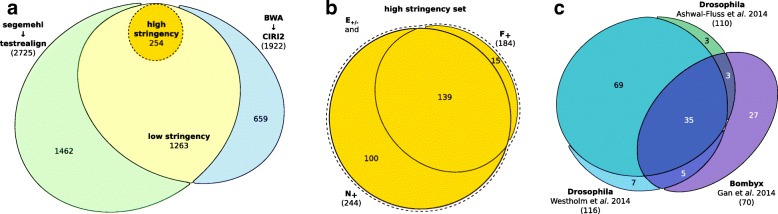
Identified circRNAs by RNA-Seq. **a** Two independent algorithms were used to predict circRNAs: segemehl in conjunction with testrealign (green) reported 2725 BSJs while BWA with CIRI2 reported 1922 BSJs. The overlap was considered as low stringency set (yellow). The high stringency set additionally requires an enrichment through RNase R treatment and compelling read coverage from at least two independent sequencing libraries. See Materials and Methods for details. These 254 candidates are most likely genuine circRNAs and are therefore used for further analysis. **b** Strong evidence is found in three independent sequencing libraries for 139 candidates. The remaining 115 are well supported by two samples. Note that all candidates were also identified as enriched in *E*_+_ vs *E*_−_. **c** 122 host genes are orthologous to host genes of circRNAs identified in either *Drosophila* or *Bombyx* in previous studies

As circRNAs do not feature 5’- or 3’-ends they are virtually resistant to RNase R treatment, which digests most linear RNAs. The enzyme can thus be used to enrich total RNA extracts for circRNAs [37, 38]. In order to identify these, we prepared RNA-Seq libraries from total RNA extracts of honeybee worker brains. The libraries were enriched for circular RNAs and compared to a non-enriched library. Each BSJ was considered as representative of a distinct circular RNA. We were able to detect a total of 3384 individual BSJs supported by at least three JSRs from the four libraries combining two different methods, see Fig. 1a. Based on these we provide two sets of circRNAs identified by applying different stringency thresholds (see Material and Methods for details). The low stringency set contains 1263 circRNAs found by both independent algorithmic methods (overlap). Only these BSJs were considered viable circRNA candidates because previous studies showed inconsistent results between different algorithms [39, 40]. Specifically, segemehl is known to produce very sensitive mapping results, potentially introducing false positives when solely relied upon. The high stringency set used for the following analyses is a subset containing 254 circRNAs with a higher amount of supporting reads along with a significant five-fold enrichment of the JSRs through RNase R treatment. The majority of the circular transcripts were even enriched by more than ten-fold (>77*%*).

A vast majority of BSJs was flanked by a canonical GT/AG splice signal. Only five circRNAs did not show such a canonical splice site (see Additional file 1). In one case an annotation was not possible. The BSJ spans two exons that are (presumably) not spliced together. The coding exon of gene CG45167 (homolog of B52 in *D. melanogaster*) and its immediate downstream exon which starts with the 5’-UTR are not present in any currently annotated transcript variant. Details are illustrated in Additional file 1.

We picked some of the most significant circRNAs that were highly abundant or showed a particularly differential expression pattern between nurse or forager bee libraries. Presence and circularity of these selected circRNAs were verified further by additional PCR experiments, see Additional file 2. TaqMan based Real-Time PCR assays were used to examine the expression levels in nurse or forager bee for two salient circRNAs in an independent experimental approach, see “Quantification in nurses and foragers”. A complete list of all 254 high confidence circRNAs including read levels and putative homologs in *D. melanogaster* and *B. mori* can be found in Additional file 3. An excerpt of the most prominent entities is shown in Table 1.

**Table 1:** Excerpt of identified circRNAs in the brain of honeybee nurse and forager bees

circRNA ID	Host gene	BeeBase	Chr.	JSRs	Enriched	Expr.	Homology
ame_circ_0001970	LOC413427	GB43145	LG11	432	6.7	0.329	⋆∘
ame_circ_0000721	LOC724885	GB53835	LG3	139	10.2	0.344	
ame_circ_0002142	LOC410393	GB52063	LG12	124	5.0	0.628	*†*
ame_circ_0000163	LOC408576	GB42249	LG1	103	7.2	0.328	⋆∘
ame_circ_0000232	Mup2	GB49259	LG1	97	11.0	0.119	
ame_circ_0001780	rad	GB49511	LG10	91	5.0	0.062	⋆∘
ame_circ_0001286	LOC411534	GB44365	LG7	63	93.0	0.731	⋆∘
ame_circ_0002579	LOC409655	GB47584	LG16	42	17.5	0.226	
ame_circ_0001822	Rsmep2	GB54272	LG10	34	5.0	0.022	⋆∘
ame_circ_0002577	LOC409655	GB47584	LG16	17	11.3	0.072	
ame_circ_0001852	CoRest	GB52614	LG10	10	5.0	0.013	⋆∘
ame_circ_0001099	LOC411114	GB44582	LG5	306	18.5	0.328	*†*
ame_circ_0000414	LOC725294	GB55364	LG2	216	7.6	0.312	⋆∘
ame_circ_0000397	LOC408688	GB49767	LG2	185	5.0	0.377	⋆∘*†*
ame_circ_0001712	LOC408996	GB42579	LG9	169	6.3	0.198	⋆∘*†*
ame_circ_0002576	LOC409655	GB47584	LG16	168	14.9	0.644	
ame_circ_0001638	LOC411347	GB17597	LG9	159	9.4	0.400	
ame_circ_0001593	LOC408991	GB53310	LG9	148	7.9	0.105	
ame_circ_0001479	LOC408957	GB40504	LG8	147	18.4	0.339	∘
ame_circ_0000524	LOC408718	GB43446	LG2	130	36.2	0.313	
ame_circ_0001120	sGC-alpha1	GB52929	LG6	129	10.4	0.276	*†*
ame_circ_0000054	LOC726544	GB42188	LG1	124	7.5	0.480	
ame_circ_0001877	LOC408309	GB45167	LG11	121	9.4	0.085	
ame_circ_0000669	LOC410044	GB55791	LG3	118	13.0	0.370	
ame_circ_0000073	LOC410717	GB55293	LG1	111	11.0	0.290	⋆∘
ame_circ_0001340	LOC411229	GB42567	LG7	109	6.9	0.570	∘

All circRNAs were significantly enriched in *E*_+_ over the non-enriched set *E*_−_. *Set* refers to the RNA-Seq libraries in which the circRNA was enriched in addition (see Table 2). The *host gene* is given according to the RefSeq GCF_000002195.4 annotation along with the corresponding *BeeBase* identifier [74]. The respective chromosome is indicated in the *Chr.* column. The summarized number of *JSR*s is given along with the averaged normalized expression levels relative to the host gene expression *expr.* and fold enrichment *enriched*. The *homology* column indicates whether a *Drosophila* or *Bombyx* homolog was found in ⋆[32] ∘[35] or *†*[34]. The first block corresponds to circRNAs that were particularly strongly expressed (many JSRs or high norm. expression) or showed signs of differential expression between nurse and forager bee libraries and were thus selected for verification of circularity and presence in further PCR experiments. The full list can be found in the Additional file 3

### Homologs to fly and silkworm

Honeybee circRNAs were compared to those found in fruit fly [32, 35] and silkworm [34] based on the homology of their parental genes (Fig. 1c). Out of 254 honeybee circRNAs only 70 host gene homologs were found in silkworm (30%). In contrast, 203 homologous BSJs were identified for fruit fly (80%) which can be explained by the closer phylogenetic relationship to honeybee [41]. Consistent with our results, circularized exons in fruit fly were found in 144 to 151 of these homologs (with respect to [32, 35], overlap 122 circRNAs). This finding is in line with a similar comparison of circRNAs in human and mouse. There, two-thirds of all host genes harboring back-splicing junctions could be correlated by homologies between the two species [42]. A complete listing of the results can be found in Additional file 3.

### GO term enrichment

A GO term analysis (gene ontology term enrichment) was performed using all 203 circRNA host gene homologs correlated to fruit fly from which we extrapolated the functional annotation. High-level processes involved in synaptic development and regulation were significantly enriched. Given that the source samples were obtained from brain tissue, this is an expected result but it also resembles the finding that neurologically associated genes are a main source of circRNAs as found for *D. melanogaster* [35]. The most enriched high level terms below a *p*-value of 10^−4^ were “anesthesia-resistant, medium- and long-term memory” (27 ×) “medium-term memory” (23 ×), “regulation of neuromuscular synaptic transmission” (21 ×) and “deactivation of rhodopsin mediated signaling” (21 ×). The former is especially remarkable. One representative of this group is the *radish* gene from which circAm*rad* (*ame_circ_0001780*) arises. We found that the abundance levels of circAm*rad* correlated with the acquired task of a bee (see “Abundance and task allocation” below). Consistent with this is also the enrichment of rhodopsin signaling and memory-related genes. Nurse bees take care of the brood inside the hive, where it is dark and the requirements to memory are different from those of foragers [43]. After task transition to forager bees, they start to collect food from outside the hive, mostly at daylight, and need to find their way back to the hive afterward. A need for adaptation of rhodopsin signaling and a change in memory requirements is obvious. In fact, “positive phototaxis” showed the highest GO term enrichment (44 ×). The significance (*p*=1.87×10^−3^) however was above the applied threshold because the term only has four representatives in the reference set. A detailed overview of enriched GO terms can be found in Additional file 4.

### Exon-intron structures

**Fig. 2:**
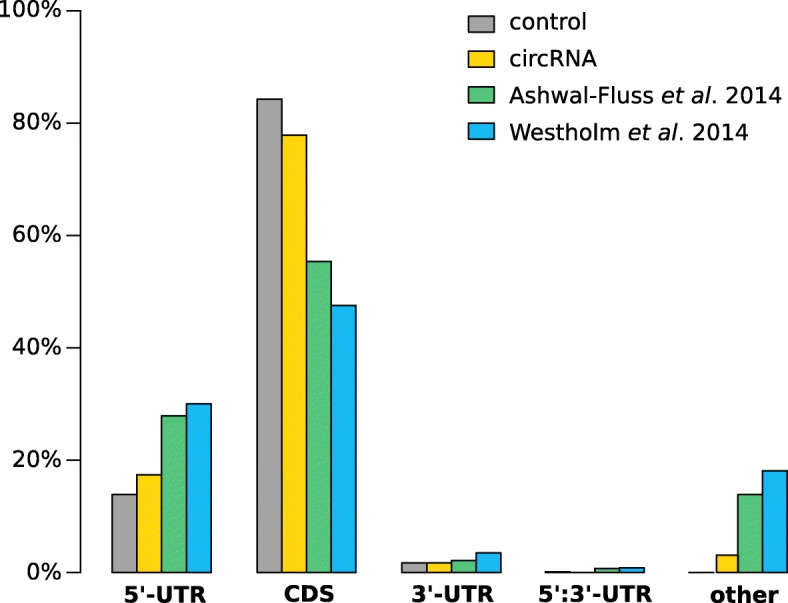
Location of circRNAs in the original transcripts. BSJs are clustered into the following categories: part of the 5’-UTR, from the coding sequence exclusively, part of the 3’-UTR, spanning from 5’ to 3’-UTR or other (in the middle of exons, introns, part of non-coding genes or in between genes). Most of the circRNAs in *A. mellifera* originate from exons in the coding portion of a transcript, which also represents the largest amount of exons transcriptome-wide. Compared to exons of a random control, circRNAs originate slightly more often from 5’-UTR exons as also described for fruit fly. However, we did not find as many BSJ including exons of the 3’-UTR as reported there. All high confidence circRNAs were mapped to annotated splice sites and we could not detect BSJ with sufficient read numbers outside of annotated regions

The majority of BSJs in honeybee corresponds exactly to exon boundaries of protein-coding regions (78%), see Fig. 2. Nearly all remaining cases are derived from 5’-UTR containing segments (17%). This is only slightly different from the set of (presumably) linearly spliced exons in the control but shows a trend towards 5’-UTRs. For both *D. melanogaster* datasets [32, 35] the overall proportion is similar but with a much stronger bias towards 5’-UTRs (∼30*%*) and non-canonical splice events, e.g. occurring in the middle of introns or exons in between genes (∼20*%*, other). The latter category was rarely found for honeybee circRNAs (<2*%*). We note that this difference might be a result of different annotation qualities for honeybee (data from 2018) and fruit fly (data from before 2014) and should thus not be over-interpreted.

**Fig. 3:**
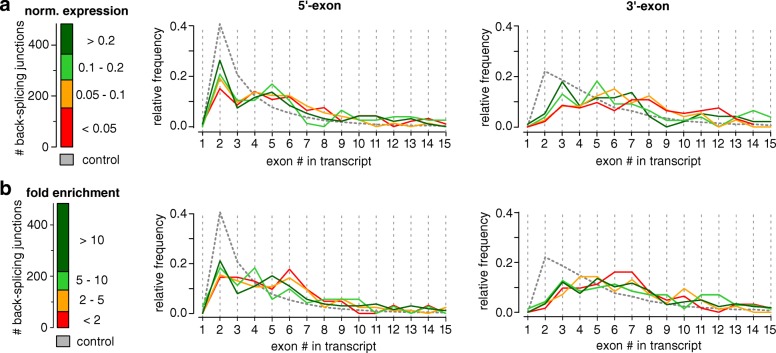
Exon position of circRNAs in the host gene. Exons of the parent transcripts were numbered. The number of the first an the last exon included in a circRNA is represented in the left (5’-end) and right (3’-end) panel, respectively. CircRNAs are stratified by (**a**) normalized circRNA expression into four groups (green to red with decreasing ratio) and (**b**) relative fold change between RNase R untreated and treated experiments (green to red with decreasing difference). As a reference, randomly selected exons in the control (gray) exhibit a higher number of second and third exons in a transcript just by chance

For fruit fly, it was reported that circRNAs mostly originate from the second exon of a transcript [35]. This is also true for honeybee circRNAs. Figure 3, however, shows that this number is implied by the outstanding abundance of transcripts with only two exons. This is also visible in the randomized control distribution. Compared to this set, the observed starts at exon two are actually less than what would be expected. We identified two factors that correlate with back-splicing: The exon position and the number of exons. The further downstream an exon is located in a transcript and the more exons (and thereby splice junctions) it exhibits, the more likely circRNAs arise from the transcript.

Another finding from fruit fly indicates that circRNAs with higher normalized expression tend to favor earlier exons than less expressed variants [35]. We reproduced this by partitioning the BSJs according to their normalized expression levels, see Fig. 3a. A similar trend is visible in our data. However, the shift towards later exons for less expressed circRNAs (e.g. with relative expression levels <0.05) is not as pronounced. Notably, our control exons exhibit a much stronger bias towards the second and third exon for the starts of circular junctions than any of the partitions with circularized exons (almost 60%), especially compared to the control used for fruit fly [35]. An alternative stratification of BSJs by their relative fold change in RNA-Seq libraries enriched for circRNAs yields the same results, see Fig. 3b. The circRNAs presented here do not involve parts of the 5’-UTR more often than expected but transcripts with unusually long 5’-UTRs appear to be prone to circularization at an increased likelihood.

### Intronic features

**Fig. 4:**
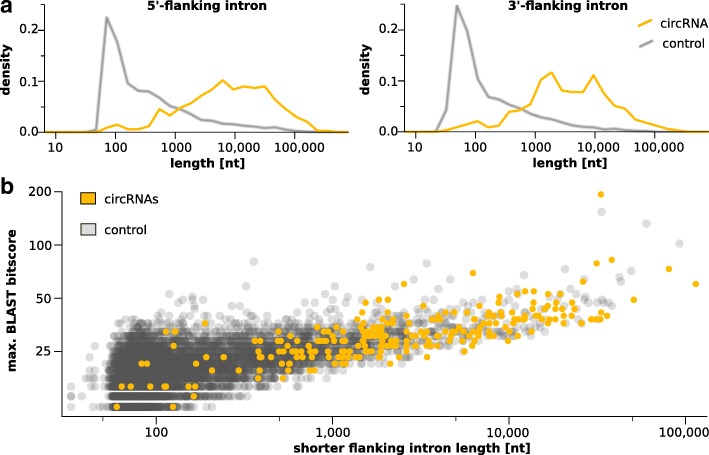
Introns flanking circRNAs. **a** Flanking introns upstream (left) and downstream (right) of circRNAs (yellow) are significantly longer (*t*-test with *p*<0.001 for both) than those of a random control (gray). **b**BLAST bitscores of the best reverse complementary match of the shorter circRNA flanking intron to the other (yellow) compared to control intron pairs (gray). Introns flanking circRNAs are visibly longer than random introns but show similar bitscores for reverse complementary matches of equal intron lengths (correlation *ρ*=0.5). Notably, shorter introns in the control frequently exhibit relatively high bitscores, indicating that short complementarity regions are common among intron pairs

**Fig. 5:**
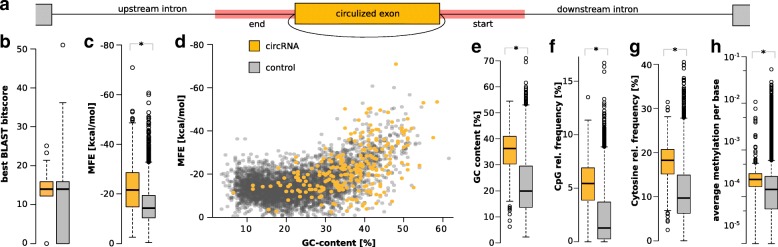
Putative interactions and sequence properties of flanking introns. **a** Scheme indicating the relevant regions (red) of introns flanking circularized exons (yellow). **b** Screen of sequence complementarity using BLAST. The scores for circular and linear control intron pairs largely overlap. **c** Screen of intron interactions represented by the MFE of constrained RNAcofold calculations. The difference between both groups is significant. Still there is some overlap. **d** Relation of GC-content and MFEs. Both measures appear to be linked (correlation *ρ*=0.5, Spearman’s rank correlation). **e** Screen of GC-content. A significant increase is found compared to the control. **f** Screen of CpG dinucleotides. A significant increase is found compared to the control which rarely exhibits CpG. **g** Screen of cytosine-content. A significant increase is found compared to the control. It explains the observed effects for GC-content and CpG frequency. **h** Screen of average base methylation. The number of methylated bases in introns flanking circRNAs is slightly but significantly increased compared to linear control introns. *p-value <0.001

In honeybee, introns flanking circularized exons are significantly longer than those from linearly spliced exons, see Fig. 4a. They can span several thousand bases. This result is in line with findings from fruit fly and human [35, 44]. There, in addition, flanking introns showed increased levels of reverse complementarity compared to linearly spliced exons. Reverse complementary regions are thought to enhance the likelihood for base-pairing between the introns. This interaction likely guides back-splicing process [36, 45, 46]. Following up on this assumption, introns were reciprocally scanned for reverse complementary matches at sequence-level using BLAST [47], see Fig. 4b. While the result shows that introns flanking circularized exons are composed of regions with better complementary (represented by higher bitscores) in general, it is also obvious that complementarity is linked to the length of introns. Higher scores of complementarity matches are likely a result of the fact that introns flanking circularized exons are much longer than those from the control set. The most relevant regions for circularization are probably the end of the 5’ flanking and the start of the 3’ flanking intron, see Fig. 5a for a scheme. Even if the comparison is limited to these regions, the difference in complementary matches cannot explain why some exons are circularized and others are not. The median complementarity is about equal to the control introns that flank linear exons even though the latter show much higher variance especially towards introns with hardly any complementarity, see Fig. 5b.

An RNA secondary structure prediction using RNAfold [48] was used to investigate potential intron-intron interactions more specifically, see Fig. 5c. The difference is more obvious using this method. Cofolded complexes of the control introns exhibit much higher minimum free energy scores (MFE), indicating less base-pairing interaction. The difference is highly significant (*p*<0.001). However, the MFE scores partly cover similar ranges, which does not allow for a clear distinction between circularized exons and linear splicing products. Figure 5d shows that the increase in folding-potential (represented by lower MFE scores) is linked to GC-content of the respective introns. Also the fact that the complementarity match, as well as the cofolding analysis, yielded similar results for all combinations of starts and ends of the flanking introns (e.g. pairing the end of the upstream intron with the end of the downstream intron) puts a direct effect of base-pairing in doubt. The GC-content in turn well discriminates circRNA introns from control introns, see Fig. 5e.

### Methylation

The intronic features raise the question, why the GC-content of circRNA flanking introns is elevated in such significant amounts (median shifted from 20 to 36%, *p*<0.001). One reasonable explanation is an increase of potential DNA-methylation at these introns (CpG islands). Figure 5f illustrates that the CpG dinucleotide frequency is also significantly increased for circRNA flanking introns and nearly absent in the control group (∼1*%*). As CpG sites are preferentially methylated [49, 50], this indicates a significant increase of potential DNA-methylation sites. Moreover, cytosine methylation and hydroxymethylation at non-CG sites (CA, CT, CC) are reported to be enriched in introns of the honeybee [51]. In line with this, Fig. 5f shows that also the cytosine mononucleotide frequency is significantly increased for circRNA flanking introns. While the genome comprises ∼16*%* cytosines, circRNA introns exhibit a median of ∼18*%* cytosines. Strikingly, the median cytosine-content of introns in the control group is as low as 10%. This can be translated into reduced methylation and hydroxymethylation potential and thereby fewer alternative splicing events for introns flanking canonically spliced RNAs compared to those that frequently result in circRNAs.

We evaluated publicly available whole-genome bisulfite sequencing data of worker bees from a previous study to comprehensively determine methylation levels [52]. Figure 5h shows that the length-normalized accumulative DNA-methylation of introns flanking circular RNAs actually tends to be increased compared to those flanking random exons. Notably, the effect was not visible using only the closest 50 or 100 nucleotides of a flanking intron but became visible using a 200 nt window or full-length introns. This is probably due to the limited windows size which is likely too small for statistical assessment.

### miRNA targets

**Fig. 6:**
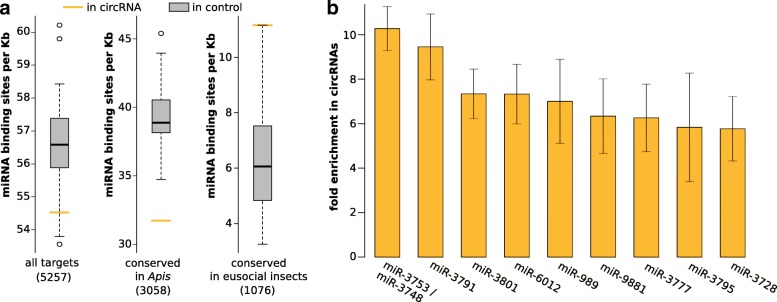
Occurrence of putative miRNA target sites in circRNA exons. **a** Putative target sites normalized by exon length in differentially conserved sets. “All targets” represents no conservation at all. *Apis* represents conservation only in closely related species. Eusocial insects are sufficiently distant to consider conservation relevant. The number of miRNA binding sites conserved in this set is significantly higher (*p*<0.001, Students *t*-test) than in the control. The absolute number of potential binding sites in the respective sets is given in parentheses below. **b** Illustration of miRNAs with potential target sites in circRNAs conserved even in eusocial insects. Only miRNAs with at least ten target sites and an at least five-fold enrichment over control are shown. Error bars indicate standard deviation in fold enrichment between different controls (*N*=42)

Potential miRNA target sites were annotated for all 254 circRNAs identified here. The results can be divided based on their degree of phylogenetic conservation. 3058 target sites were only conserved in *Apis* species. We argue that *Apis* species are too closely related to qualify as a reliable predictor for miRNA target sites. The sequence conservation in this set appears rather high in general. This is also reflected by a similar distribution of potential miRNA target sites compared to the control without any constraints on conservation, see Fig. 6a.

A set of 1076 sites is conserved in *Apis* and eusocial insects which are sufficiently distant to *A. mellifera* to reasonably infer conservation. With about 10.4 target sites per 1000 nt circRNAs have a 1.7 × increase in conserved, putative miRNA target sites compared to the median of the linear splice product control. Thus, in line with previous findings for *Drosophila* [35], we report a general enrichment of conserved miRNA target sites in circRNAs over random linear counterparts. The most enriched miRNA target sites correspond to ame-miR-3748/ame-miR-3753 (∼10× enriched, same seed region) and ame-miR-3791 (∼9.2× enriched), see Fig. 6b. RNA expression studies show that the abundance levels of some miRNAs correlate with task or age of honeybees [25–27, 53]. We did, however, not find a significant overlap of miRNAs corresponding to enriched target sites and miRNAs reported as differentially expressed in nurses and foragers. The complete list of potential target sites and their degree of conservation can be found in Additional file 5.

### Quantification in nurses and foragers

**Fig. 7:**
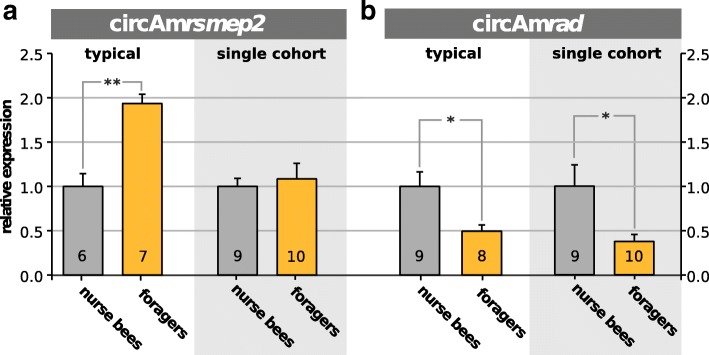
Expression analysis. TaqMen expression analysis of (**a**) circAm*rsmep2* and (**b**) circAm*rad* in brains of nurse bees and foragers from colonies with a typical age structure and SCCs consisting of bees of the same age. The expression is given relative to nurse bees. Bars show relative mean expression with standard error. The number of biological replicates is indicated in the bars. Significant differences are indicated (* *p*<0.05, ** *p*<0.01, Two-tailed unpaired Students *t*-test). In contrast to circAm*rsmep2* the expression of circAm*rad* seems correlated with the allocated task rather than the bees’ age

The circRNAs *ame_circ_0001780* and *ame_circ_0001822* showed a notable differential expression pattern in RNA-Seq results of nurse bees and foragers. For simplicity they will be termed according to their host genes in the further course of the study: circAm*rad* and circAm*rsmep2*, respectively. As the experimental setup is not suitable for any reliable quantitative assertions, we decided to perform a targeted quantitative Real-Time PCR for these candidates at different developmental stages. In addition, we compared the expression patterns in bees with age-related task allocation to those undergoing a task allocation due to colony needs (same-age, SCC), see Fig. 7.

For circAm*rsmep2* we found that expression in the brain is higher in foragers than in nurse bees (Fig. 7a). This difference, however, does not seem to be directly task-related. In a SCC where the nurse and forager bees have exactly the same age, no expression differences are observed (Fig. 7a). Our interpretation is that this expression difference most likely depends on the bees’ age but not on its task.

In contrast, circAm*rad* is higher expressed in brains of nurse bees than in brains of foragers (Fig. 7b, typical). Strikingly, this is inversely correlated with the expression of the linear product which is strongly increased in foragers (XM_393494.2/Am*rad*, *log*_2_ *ratio*∼6.1) [18] and holds true independent of the age-related task transition. The expression levels in the SCC experiment (Fig. 7b, single cohort) are similar to that of typical colonies where tasks are allocated based on a bee’s age. This data suggests a correlation of acquired task and circAm*rad* levels.

## Discussion

We remark that all circRNAs reported here are expressed in the brain of nurse and forager bees. In contrast, 2513 circRNAs reported for *D. melanogaster* [35] and 3916 for *B. mori* [34] are based on samples of different developmental stages, tissues and even cultured cells and do not ensure RNase R enrichment. All analyses were performed using the high stringency set which requires a notable amount of supporting JSRs along with a significant enrichment through RNase R treatment. We argue that the enrichment control is necessary to discern genuine circRNAs from potential trans-splicing or exon-shuffling events. Otherwise independent experiments would be required to further support the sequencing-based evidence which is not feasible given the high number of involved loci. Focussing on this significantly expressed subset of circRNAs allows us to investigate genomic properties that are an inherent part of circRNA deriving loci. The inclusion of candidates with less confidence (less enrichment, fewer supporting JSRs) would introduce additional noise into statistical analyses. This observation was made e.g. regarding lower read numbers in a *D. melanogaster* study [32]. For the majority of circRNAs identified here, the amount of canonically spliced transcripts (linear) is at least the same as the amount of back-spliced transcripts (circular). For this reason, it is unlikely that the circRNAs presented here arose from a mapping artifact, e.g. due to misalignment of reads or repeating gene copies.

Even though the exact mechanism is unknown so far, DNA-methylation is known to induce alternative splicing in honeybees [54, 55]. Our results indicate that this might affect the formation of circRNAs as well. A potential connection to the age and the allocated task is given by the fact that methylation patterns also vary depending on the age and allocated task of an individual bee [49, 50, 56]. It was even shown that reverted nurse bees regain their original methylation patterns independent of their age [52]. To follow up on this hypothesis, we correlated data from a methylation study [52] with our annotation of circRNAs. While relevant social roles are comparable, we note that collection times and extraction methods differ from experiments done in this study. Ideally, the libraries used for circRNA detection and DNA-methylation analysis should be derived from the same biological sample. Without further experimental investigation, a strong conclusion cannot be drawn yet. We argue, however, that the data presented here provides first indications for a link of circularization and DNA-methylation in honeybees. On this basis, we speculate that the age-depended increase of circRNA abundance is not (only) due to potentially lower decay rates of circRNAs compared to linear products but also a result of increasing DNA-methylation that leads to alternative splicing accompanied by an increase of circRNA formation.

The quantification of circAm*rsmep2* in nurses and foragers indicates that its abundance most likely depends on the bees’ age but not on its task. Supposedly, circAm*rsmep2* accumulates over time in the brain of worker bees, as shown for certain circRNAs in the nervous system from mammals to flies [35, 57]. On the other hand, a significant increase of the linear product in foragers was reported previously (XM_393489.3/Am*rsmep2*, *log*_2_ *ratio*∼2.8) [18]. The observed increase of the circular product circAm*rsmep2* might thus be a consequence of generally increased expression of the host gene, which codes for a RIM-family (Rab3a-interacting molecule) protein. Studies in Tetrapoda species (human, mouse, chicken and so on) show that this family plays an important role in neuronal plasticity, especially in neurotransmitter release and in organizing active zones in plasma membranes [58, 59].

Contrary to this, the data for circAm*rad* suggests a correlation of acquired task and circAm*rad* levels. Either the task of the bee is influencing circAm*rad* expression or *vice versa*. Its host gene is orthologous to the *radish* gene in *D. melanogaster*, which is known to play a crucial role in the amnesia-resistant memory (ARM). Unlike the long-term memory ARM does not require protein *de novo* synthesis [60] and thus represents a low costs memory form [61, 62]. *Rad* also exhibits circRNAs in the fly (Table 1), but whether this circRNA is involved in ARM or whether ARM is also present in honeybees, has not yet been investigated.

Studying circRNAs in the context of synaptic plasticity and neuronal processes promises further insights into the mechanism of task allocation and behavioral regulation of honeybees and probably also of other insects. New evolving techniques such as genome editing using CRISPR/Cas9 which is also available in honeybees [63] and the microinjection of short interfering RNAs into the *medial ocellus* [64] will be promising approaches to study the physiological and behavioral effects of altered circRNA levels. The latter could be used to decrease circRNA levels in the brain by specifically targeting circular junctions and thereby promoting their decay. Genome editing, on the other hand, might provide means to induce changes to introns that alter the formation of circRNAs.

## Conclusion

In total, 1263 RNAs were identified in the brain of honeybees (*A. mellifera*) using RNA-Seq. This social model organism complements previous studies in *D. melanogaster* and *B. mori*. Given the evolutionary relationship of the three species, the amount of homologous host genes with circular products, and the number of putative miRNA targets, it can be assumed that circRNAs are a common feature among insects.

Back-splicing in honeybee occurs preferentially towards the end of transcripts and in transcripts with a high number of exons. As reported for *D. melanogaster*, back-splicing is correlated with the length of the 5’ and 3’ flanking introns [35]. Additionally, a correlation was found regarding the cofolding probability of these intronic regions as well as their CpG- and cytosine-contents which might be relevant for DNA-methylation. In fact, the methylation was found to be increased for circRNA-flanking introns.

A number of circRNAs identified here were confirmed in independent PCR experiments. Two circRNAs showed a differential expression in nurse and forager bees. While circAm*rsmep2* seems to accumulate with age, the expression of circAm*rad* correlates with the performed task. This finding is the first indication of a link between the circRNA and the social role of honeybees. In general, an increase in phototaxis- and memory-related host genes for circRNAs was found.

## Methods

### Collection of bees

Bees were derived from colonies with normal age structure and with a naturally mated queen located on the grounds of the University of Würzburg. Bees were considered as nurse bees if they clearly poked their head into open brood cells containing young larvae. Foragers were captured when returning from a foraging flight and having huge pollen loads at their hind legs. Collected bees were frozen in liquid nitrogen immediately. A single cohort colony was established by transferring 2500 newly emerged bees (marked by the same color immediately after hatching) into a small hive together with one queen in one brood frame and one frame with pollen and honey. Single cohort colony bees were collected at the age of eleven days and controlled for their social task. For details regarding biological replicates in the RNA-sequencing analysis and quantitative Real-Time PCR, refer to the respective sections below.

### RNA-Seq

We used a total of four RNA-Seq libraries to determine circular transcripts present in the brain of honeybees. First, an enrichment control was compiled from the brains of ten dissected nurse bees and ten dissected foragers. Total RNA was extracted with Isol-RNA lysis reagent (5PRIME, Hilden, Germany) and treated with DNase I. The sample was divided into two halves. One half (*E*_+_) was treated with 3 units RNase R (epicentre, Madison, USA) per *μ*g total RNA. Digestion was performed for 30 min at 37 °C. For the other half (*E*_−_) an equivalent volume of double distilled water was added. Afterward, both samples were purified using phenol–chloroform extraction. Efficacy of the RNase R treatment was verified in a control experiment shown in Additional file 6. Second, we took additional samples from ten nurses and ten foragers separately and treated both with RNase R as described above (samples *F*_+_ and *N*_+_, respectively) in order to distinguish task dependent expression levels. In sum, three enriched libraries were independently generated. Only results which were confirmed in at least two libraries (considered as biological replicates) are reported. The RNA quality was ensured using an Agilent 2100 Bioanalyzer. Quality measurements, library preparation and Illumina^Ⓒ^ sequencing (125 nt paired-end) were performed by GATC Biotech AG (Konstanz, Germany). All RNA-Sequencing data was made publicly available via bioproject PRJNA345404, see Table 2.

**Table 2:** Summary of RNA-Seq libraries published along with this study

Sample	SRA ID	Role	Treatment	# Reads
*E*	SRR4343845	Both	-	8,432,479
*E*_+_	SRR4343846	Both	RNase R	7,690,777
*N*_+_	SRR4343847	Nurse	RNase R	5,843,829
*F*_+_	SRR4343848	Forager	RNase R	5,931,097

Samples were taken from brains of nurse bees, forager bees or a blend of both

### Identification of circular RNAs

We used two independent algorithmic approaches for the identification of circular RNAs. In one approach reads were mapped to the NCBI *A. mellifera* genome version 4.5 release 102 (RefSeq GCF_000002195.4) using segemehl (v0.2.0) with the split reads option [65]. The alignment was subsequently screened for model-free splicing events using the accompanied testrealign tool. In the second approach we used BWA (v0.7.5a) as mapping tool and subsequently screened using CIRI2 (v2.0.6) with default parameters [66, 67]. Identified junctions were post-processed using custom scripts bundled in our Chiasm suite. Chiasm was also used to perform the statistical calculations later on (e.g. CpG-content, pairing-probability, see below). The full analysis pipeline is publicly available at https://git.io/chiasm. More precisely, junctions with almost identical start and end positions were merged if they differed by less than 6 nt. Junctions mapped +/-5 nt next to exon boundaries were corrected to exactly match the boundary. This accounts for small variations in sequencing and mapping, e.g. due to flanking intron sequence being potentially identical to the junctioning exon or indels in the genome. We assigned the respective gene and exon numbers to each hit and normalized the number of JSR to the host gene’s total read number. Analogously to present studies in *Drosophila* [35] we normalized BSJ read counts (*norm*(*n*_∘_)) by dividing the number or circular JSRs (*n*_∘_) by the number of mapped library reads *N* (in millions), divided by reads per kilobases in million reads (RPKM) of the host gene (*g*). The latter is defined as number or reads assigned to the host gene (*n*_*g*_) divided by the length of the gene (*l*_*g*_) in thousand bases and divided by library size of mapped reads, *N*, in millions.

1 $$ norm\left(n_{\circ}\right) = \frac{n_{\circ}}{\frac{N}{1,000,000} ~ RPKM_{g}}  $$

with

2 $$ RPKM_{g} = \frac{n_{g}}{\frac{l_{g}}{1,000} ~ \frac{N}{1,000,000}}  $$

We divided the identified circular RNAs into two sets limited by different stringency levels. The low stringency set contains all circRNAs picked up by both approaches (testrealign and CIRI2) with at least three JSRs. In the high stringency set, we only considered BSJs with more than ten JSRs across all libraries as suggested in literature [35]. Thereby, the BSJ has to be found in library *E*_+_ and at least one other independent RNase R treated library. Moreover, a five-fold enrichment of JSRs in the RNase R treated library (*E*_+_ vs *E*_−_) is required.

### Validation of circRNAs

Total RNA was extracted from ten worker bee brains and prepared as described for the RNA-Seq preparation (see above, without enrichment by RNase R). After DNA digestion, 1 *μ*g of RNA was transcribed into cDNA using RevertAid H minus reverse transcriptase (ThermoFisher Scientific) adhering to the manufacturer’s specifications. For PCR amplification 15 *μ*mol of divergent primers were added to 10 ng of cDNA with 25 *μ*L of Phusion Polymerase master mix. PCR steps were 30 sec heating to 98 °C followed by 35 cycles of 10 sec denaturation at 98 °C, 10 sec annealing at 62 °C and 8 sec elongation at 72 °C. After a final extension period of 10 min at 72 °C, PCR products were either stored at -20 °C or subjected to agarose gel electrophoresis. Primer sequences are provided in Additional file 7. The results of PCR verification are provided in Additional file 2.

### Quantification of circRNAs

750 *μ*L of Isol-RNA lysis reagent (5PRIME, Hilden, Germany) was added to frozen brain samples and homogenized subsequently. After adding 150 *μ*L of chloroform and consequent phase separation the aqueous phase was transferred to 900 *μ*L ethanol (75%). RNA was purified using peqGOLD Total RNA Kit (Peqlab, Erlangen, Germany) following the standard protocol provided by the manufacturer including an optional DNase I digestion step. From each bee 1.5 *μ*g of total brain RNA was transcribed using qScriber cDNA Synthesis Kit (highQu, Kraichtal, Germany). Triplicates of each cDNA (5 *μ*L) were run in a quantitative Real-Time PCR on a Rotor-Gene Q (Qiagen, Hilden, Germany) in a total reaction volume of 25 *μ*L, containing each primer (0.25 *μ*M), TaqMan probe (0.1 *μ*M), Rotor-Gene Multiplex PCR 9Master Mix (Qiagen, Hilden, Germany). The number of biological replicates is specified in Fig. 7. TaqMan probe sequences are provided in Additional file 7. The following protocol was used: 60 °C for 1 min, 95 °C for 5 min and 45 cycles at 95 °C for 20 sec and 60 °C for 1 min. Afterward the relative expression to AmEF1 *α* [68] with the *ΔΔC*_*t*_ method was determined using Rotor-Gene Q software (Qiagen, Chatsworth, CA). Expression of circRNA was compared only if respective groups did not differ in their AmEF1 *α* expression (*p*>0.05, Student’s t-test). For the circRNA candidates, circAm*rsmep2* and circAm*rad* the established TaqMan probe-based assays were designed using outward facing primers. PCR experiments for detection of circRNAs were designed analogously to [30]. The TaqMan probe binds directly to the circular junction and thus signals can only derive from non-canonical spliced RNAs.

### Homology screen and functional annotation

Predicted circRNAs were correlated to those previously reported for *D. melanogaster* [32, 35] and *B. mori* [34]. We matched the loci based on the predicted homologs of the closest protein-coding gene with respect to OrthoDB v9 [69]. CircRNAs from genes without homolog could thus not be accounted for. Homologous fruit fly genes were then submitted to the online PANTHER annotation platform for further over-representation analysis using Fisher’s Exact test with false discovery rate (FDR) multiple testing correction. We included functional annotations with more than five-fold over-representation and FDR below 1%.

### Sequence and structural analysis

Based on the genomic annotation and the largest spanning transcript of each circRNA that contained exon boundaries, we extracted whether the circRNA contained part of the 5’-UTR, 3’-UTR of a canonical protein-coding transcript or if it exclusively contained coding regions. The number of exons spanned by the transcript was noted for the 5’ and 3’ end of the BSJ. For comparison to potentially non-circular transcripts, a random control was generated by drawing genes with more than two exons proportionally from all chromosomes and picking exon boundary pairs that were neither from the start nor the end of the transcript. Genes harboring any JSRs found in this study were excluded from this control, see Additional file 3. A random control of 10,000 such junctions was generated for all following statistical tests. Flanking introns were determined by including the sequence outside of the BSJ until the next exon in the same transcript.

In order to screen for complementarity between flanking intron pairs, the 5’ intron was matched to the 3’ intron using BLAST [47] with a word size of six to determine the highest scoring stretch of reverse complementarity. We repeated the procedure with 100 nt from the end of the upstream and 100 nt from the start of the downstream intron, to discern whether approximate regions showed increased complementarity. The same 100 nt portions were used for structural analysis utilizing RNAcofold [48]. We applied soft constraints to ensure MFE scores solely based on base-pairing between both intronic regions. Both procedures were repeated with all combinations of starts and ends of the respective introns as educated control set (an interaction of the end of the upstream and the end of the downstream intron is probably not relevant). Surprisingly, the results for all combinations were similar. To rule out, that we bias for specific length effects at 100 nt, all calculations were also done with 50 and 200 nt without changing the outcome (data not shown). Introns were checked for GC-content ignoring undetermined residues in the genome sequence (N). Similarly, the mononucleotide frequency of cytosine and the relative frequency of CpG dinucleotides was calculated.

To asses, whether the observed increase of potential DNA-methylation sites is reflected in actual DNA-methylation, we used whole genome bisulfite sequencing data of worker bees that was publicly available. Precisely, we used all native worker libraries provided in BioProject PRJNA104931 [52] and combined them for this analysis as no differences in average methylation was found between nurse and forager bee libraries for the genes relevant in this study (data not shown). Methylation patterns were analyzed using Bismark [70] v0.19.1 with Bowtie2 [71] v2.2.6 for bisulfite specific mapping and default parameters suggested by its authors. For each intron, we counted the average methylation per base on both strands. An average coverage of at least five reads for each intron was required. Calculations were done for 50, 100 and 200 nt as well as for the length of the complete intron where it exceeded 200 nt and numbers were normalized by the respective sequence length. A single-sided Wilcoxon-Mann-Whitney rank-sum test was used to determine the significance of the increase over the control.

### miRNA interference analysis

Predicted and experimentally verified miRNA sequences of *A. mellifera* were obtained from miRBase [72] release 21. Potential target sites were screened in all exon sequences overlapping with the identified circRNAs using nucleotide two to seven of the mature miRNA sequence, see [73]. The analysis pipeline is publicly available on git.io, see above. For each potential miRNA binding site, we determined conservation in further *Apis* species (*A. cerana*, *A. dorsata*, *A. florea*) and other eusocial insects (*E. dilemma*, *L. ventralis*, *M. quadrifasciata*, *B. impatiens*, *B. terrestris*) for the seed region with 100 nt up- and downstream using the best BLAST match [47] in the respective genome. We considered a site conserved if the 6 nt seed region was perfectly conserved among three out of four *Apis* or four out of five eusocial insects, respectively. As a random control we used linear exons, see “Sequence and structural analysis”. We split the control to sets of about equal size (42 sets) and applied the above procedure to each set. This results in 42 control datasets where each represents a subset of exons with similar length to avoid a bias due to an over-representation of certain length species. Identified target sites were normalized to sites per 1000 nt.

## Additional files

Additional file 1 Splicing. PDF showing the splice site motif of circRNAs and details on exceptions. (PDF 547 kb)

Additional file 2 Verification of circRNAs via PCR. Results of the circularity validation through PCR. (PDF 582 kb)

Additional file 3 List of circRNAs in honeybee identified here. Excel table of all circRNAs identified here. The data is presented analogous to Table 1 but addressing additional information and details for all RNA-Seq libraries. A second sheet contains a similar list including all 3384 circRNAs identified by both algorithmic approaches based on JSRs in any library. (XLSX 411 kb)

Additional file 4 GO term enrichment. Excel table showing the results of a GO term enrichment analysis for the host genes of circRNAs based on homologous fruit fly genes performed with the PANTHER annotation platform. Terms with at least five-fold over-representation and a false discovery rate (FDR) below 1% were considered. From these, we limited the interpretation with a relevant *p*-value threshold of 10^−4^ which is marked in the table. (XLSX 12 kb)

Additional file 5 List of potential miRNA targets including conservations. Excel table of all miRNA target sites found on circRNA sequences identified here. It contains detailed data on the target circRNA, the potential position of interaction and its conservation in *Apis*, eusocial insects, *Drosophila* and *Bombyx*. (XLSX 231 kb)

Additional file 6 RNase R enrichment control. Experimental control of circRNA enrichment over linear products. (PDF 86 kb)

Additional file 7 List of primers and probes for PCR and TaqMan assay. Excel table with a list of all PCR primers used in this study. A second sheet lists the TaqMan probes. (XLSX 10 kb)

## Abbreviations

BSJ: Back-spliced junction
circRNA: Circular RNA
cDNA: Complementary DNA
C _*t*_: Threshold cycle
FDR: False discovery rate
GO: Gene ontology
ID: Identifier
JSR: Junction-spanning read
MFE: Minimum free energy
miRNA: Micro RNA
PCR: Polymerase chain reaction
RNA-Seq: RNA-sequencing
SCC: Single cohort colony
SRA: Sequence read archive
UTR: Untranslated region

## References

[1] Oster GF, Wilson EO (1978). Caste and ecology in the social insects. Monogr Popul Biol.

[2] Dreller C, Page Jr. ER, Fondrk KM (1999). Regulation of pollen foraging in honeybee colonies: effects of young brood, stored pollen, and empty space. Behav Ecol Sociobiol.

[3] Le Conte Y, Mohammedi A, Robinson GE (2001). Primer effects of a brood pheromone on honeybee behavioural development. Proceed Biol Sci.

[4] Vaughan MD, Calderone WN (2002). Assessment of pollen stores by foragers in colonies of the honey bee, Apis mellifera l. Insect Soc.

[5] Robinson GE, Page RE, Strambi C, Strambi A (1989). Hormonal and genetic control of behavioral integration in honey bee colonies. Sci (New York, N.Y.).

[6] Seeley TD (1995). The Wisdom of the Hive.

[7] Thamm M, Scheiner R (2014). Pkg in honey bees: spatial expression, amfor gene expression, sucrose responsiveness, and division of labor. J Comp Neurol.

[8] Scheiner R, Reim T, Søvik E, Entler BV, Barron AB, Thamm M (2017). Learning, gustatory responsiveness and tyramine differences across nurse and forager honeybees. J Exp Anim Sci.

[9] Değirmenci L, Thamm M, Scheiner R. Responses to sugar and sugar receptor gene expression in different social roles of the honeybee (Apis mellifera). J Insect Physiol. 2017. 10.1016/j.jinsphys.2017.09.009. 10.1016/j.jinsphys.2017.09.009 28935437

[10] Withers GS, Fahrbach SE, Robinson GE (1993). Selective neuroanatomical plasticity and division of labour in the honeybee. Nature.

[11] Fahrbach SE, Moore D, Capaldi EA, Farris SM, Robinson GE (1998). Experience-expectant plasticity in the mushroom bodies of the honeybee. Learn Mem (Cold Spring Harbor, N.Y.).

[12] Durst C, Eichmüller S, Menzel R (1994). Development and experience lead to increased volume of subcompartments of the honeybee mushroom body. Behav Neural Biol.

[13] Scholl C, Wang Y, Krischke M, Mueller MJ, Amdam GV, Rößler W (2014). Light exposure leads to reorganization of microglomeruli in the mushroom bodies and influences juvenile hormone levels in the honeybee. Dev Neurobiol.

[14] Farris SM, Robinson GE, Fahrbach SE (2001). Experience- and age-related outgrowth of intrinsic neurons in the mushroom bodies of the adult worker honeybee. J Neurosci.

[15] Groh C, Ahrens D, Rössler W (2006). Environment- and age-dependent plasticity of synaptic complexes in the mushroom bodies of honeybee queens. Brain Behav Evol.

[16] Muenz TS, Groh C, Maisonnasse A, Le Conte Y, Plettner E, Rössler W (2015). Neuronal plasticity in the mushroom body calyx during adult maturation in the honeybee and possible pheromonal influences. Dev Neurobiol.

[17] Kucharski R, Maleszka R (2002). Evaluation of differential gene expression during behavioral development in the honeybee using microarrays and northern blots. Genome Biol.

[18] Liu F, Li W, Li Z, Zhang S, Chen S, Su S (2011). High-abundance mRNAs in Apis mellifera: comparison between nurses and foragers. J Insect Physiol.

[19] Lutz CC, Rodriguez-Zas SL, Fahrbach SE, Robinson GE (2012). Transcriptional response to foraging experience in the honey bee mushroom bodies. Dev Neurobiol.

[20] Whitfield CW, Ben-Shahar Y, Brillet C, Leoncini I, Crauser D, Leconte Y, Rodriguez-Zas S, Robinson GE (2006). Genomic dissection of behavioral maturation in the honey bee. Proc Natl Acad Sci.

[21] Han B, Fang Y, Feng M, Hu H, Qi Y, Huo X, Meng L, Wu B, Li J (2015). Quantitative neuropeptidome analysis reveals neuropeptides are correlated with social behavior regulation of the honeybee workers. J Proteome Res.

[22] Han B, Fang Y, Feng M, Hu H, Hao Y, Ma C, Huo X, Meng L, Zhang X, Wu F, Li J (2017). Brain membrane proteome and phosphoproteome reveal molecular basis associating with nursing and foraging behaviors of honeybee workers. J Proteome Res.

[23] Bezabih G, Cheng H, Han B, Feng M, Xue Y, Hu H, Li J (2017). Phosphoproteome analysis reveals phosphorylation underpinnings in the brains of nurse and forager honeybees (apis mellifera). Sci Rep.

[24] Han B, Fang Y, Feng M, Lu X, Huo X, Meng L, Wu B, Li J (2014). In-depth phosphoproteomic analysis of royal jelly derived from western and eastern honeybee species. J Proteome Res.

[25] Behura SK, Whitfield CW (2010). Correlated expression patterns of microRNA genes with age-dependent behavioural changes in honeybee. Inst Mol Biol.

[26] Liu F, Peng W, Li Z, Li W, Li L, Pan J, Zhang S, Miao Y, Chen S, Su S (2012). Next-generation small RNA sequencing for microRNAs profiling in Apis mellifera: comparison between nurses and foragers. Inst Mol Biol.

[27] Weaver DB, Anzola JM, Evans JD, Reid JG, Reese JT, Childs KL, Zdobnov EM, Samanta MP, Miller J, Elsik CG (2007). Computational and transcriptional evidence for microRNAs in the honey bee genome. Genome Biol.

[28] Memczak S, Jens M, Elefsinioti A, Torti F, Krueger J, Rybak A, Maier L, Mackowiak SD, Gregersen LH, Munschauer M, Loewer A, Ziebold U, Landthaler M, Kocks C, le Noble F, Rajewsky N (2013). Circular RNAs are a large class of animal RNAs with regulatory potency. Nature.

[29] Shen T, Han M, Wei G, Ni T (2015). An intriguing RNA species–perspectives of circularized rna. Protein Cell.

[30] Jeck WR, Sharpless NE (2014). Detecting and characterizing circular RNAs. Nat Biotechnol.

[31] Salzman J, Chen RE, Olsen MN, Wang PL, Brown PO (2013). Cell-type specific features of circular RNA expression. PLoS Genet.

[32] Ashwal-Fluss R, Meyer M, Pamudurti NR, Ivanov A, Bartok O, Hanan M, Evantal N, Memczak S, Rajewsky N, Kadener S (2014). circRNA biogenesis competes with pre-mRNA splicing. Mol Cell.

[33] Hansen TB, Jensen TI, Clausen BH, Bramsen JB, Finsen B, Damgaard CK, Kjems J (2013). Natural RNA circles function as efficient microRNA sponges. Nature.

[34] Gan H, Feng T, Wu Y, Liu C, Xia Q, Cheng T (2017). Identification of circular RNA in the Bombyx mori silk gland. Insect Biochem Mol Biol.

[35] Westholm JO, Miura P, Olson S, Shenker S, Joseph B, Sanfilippo P, Celniker SE, Graveley BR, Lai EC (2014). Genome-wide analysis of drosophila circular RNAs reveals their structural and sequence properties and age-dependent neural accumulation. Cell Rep.

[36] Kramer MC, Liang D, Tatomer DC, Gold B, March ZM, Cherry S, Wilusz JE (2015). Combinatorial control of Drosophila circular RNA expression by intronic repeats, hnrnps, and sr proteins. Genes Dev.

[37] Suzuki H, Zuo Y, Wang J, Zhang M., Malhotra A, Mayeda A (2006). Characterization of RNase R-digested cellular RNA source that consists of lariat and circular RNAs from pre-mRNA splicing. Nucleic Acids Res.

[38] Vincent HA, Deutscher MP (2006). Substrate recognition and catalysis by the exoribonuclease RNase R. J Biol Chem.

[39] Zeng X, Lin W, Guo M, Zou Q (2017). A comprehensive overview and evaluation of circular RNA detection tools. PLoS Comput Biol.

[40] Shen Y, Guo X, Wang W (2017). Identification and characterization of circular RNAs in zebrafish. FEBS Lett.

[41] Wiegmann BM, Trautwein MD, Kim J-W, Cassel BK, Bertone MA, Winterton SL, Yeates DK (2009). Single-copy nuclear genes resolve the phylogeny of the holometabolous insects. BMC Biol.

[42] Guo JU, Agarwal V, Guo H, Bartel DP (2014). Expanded identification and characterization of mammalian circular RNAs. Genome Biol.

[43] Naeger NL, Van Nest BN, Johnson JN, Boyd SD, Southey BR, Rodriguez-Zas SL, Moore D, Robinson GE (2011). Neurogenomic signatures of spatiotemporal memories in time-trained forager honey bees. J Exp Bio.

[44] Jeck WR, Sorrentino JA, Wang K, Slevin MK, Burd CE, Liu J, Marzluff WF, Sharpless NE (2013). Circular RNAs are abundant, conserved, and associated with alu repeats. RNA (New York, N.Y.).

[45] Liang D, Wilusz JE (2014). Short intronic repeat sequences facilitate circular RNA production. Genes Dev.

[46] Starke S, Jost I, Rossbach O, Schneider T, Schreiner S, Hung L-H, Bindereif A (2015). Exon circularization requires canonical splice signals. Cell Rep.

[47] Camacho C, Coulouris G, Avagyan V, Ma N, Papadopoulos J, Bealer K, Madden TL (2009). Blast+: architecture and applications. BMC Bioinform.

[48] Lorenz R, Bernhart SH, Zu Siederdissen CH, Tafer H, Flamm C, Stadler PF, Hofacker IL (2011). ViennaRNA Package 2.0. Algoritm Mol Biol.

[49] Oka M, Rodić N, Graddy J, Chang L-J, Terada N (2006). CpG sites preferentially methylated by Dnmt3a in vivo. J Biol Chem.

[50] Lyko F, Foret S, Kucharski R, Wolf S, Falckenhayn C, Maleszka R (2010). The honey bee epigenomes: differential methylation of brain DNA in queens and workers. PLoS Biol.

[51] Cingolani P, Cao X, Khetani RS, Chen C-C, Coon M, Sammak A, Bollig-Fischer A, Land S, Huang Y, Hudson ME, Garfinkel MD, Zhong S, Robinson GE, Ruden DM (2013). Intronic non-CG DNA hydroxymethylation and alternative mRNA splicing in honey bees. BMC Genomics.

[52] Herb BR, Wolschin F, Hansen KD, Aryee MJ, Langmead B, Irizarry R, Amdam GV, Feinberg AP (2012). Reversible switching between epigenetic states in honeybee behavioral subcastes. Nat Neurosci.

[53] Chen H, Li Y, Chen K, Yao Q, Li G, Wang L (2010). Comparative proteomic analysis of Bombyx mori hemolymph and fat body after calorie restriction. Acta Biochim Pol.

[54] Shukla S, Kavak E, Gregory M, Imashimizu M, Shutinoski B, Kashlev M, Oberdoerffer P, Sandberg R, Oberdoerffer S (2011). CTCF-promoted RNA polymerase II pausing links DNA methylation to splicing. Nature.

[55] Li-Byarlay H, Li Y, Stroud H, Feng S, Newman TC, Kaneda M, Hou KK, Worley KC, Elsik CG, Wickline SA, Jacobsen SE, Ma J, Robinson GE (2013). RNA interference knockdown of DNA methyl-transferase 3 affects gene alternative splicing in the honey bee. Proc Natl Acad Sci.

[56] Becker N, Kucharski R, Rössler W, Maleszka R (2016). Age-dependent transcriptional and epigenomic responses to light exposure in the honey bee brain. FEBS Open Bio.

[57] Rybak-Wolf A, Stottmeister C, Glažar P, Jens M, Pino N, Giusti S, Hanan M, Behm M, Bartok O, Ashwal-Fluss R, Herzog M, Schreyer L, Papavasileiou P, Ivanov A, Öhman M, Refojo D, Kadener S, Rajewsky N (2015). Circular RNAs in the mammalian brain are highly abundant, conserved, and dynamically expressed. Mole Cell.

[58] Gandini MA, Felix R (2012). Functional interactions between voltage-gated Ca 2+ channels and Rab3-interacting molecules (RIMs): New insights into stimulus–secretion coupling. Biochim Biophys Acta Biomembr.

[59] Garner CC, Kindler S, Gundelfinger ED (2000). Molecular determinants of presynaptic active zones. Curr Opin Neurobiol.

[60] Folkers E, Waddell S, Quinn WG. Proceedings of the National Academy of Sciences of the United States of America. 2006; 103(46):17496–00. 10.1073/pnas.0608377103. 10.1073/pnas.0608377103 PMC1634833 17088531

[61] Mery F, Kawecki TJ (2005). A cost of long-term memory in Drosophila. Sci (New York, N.Y.).

[62] Tully T, Preat T, Boynton SC, Del Vecchio M (1994). Genetic dissection of consolidated memory in Drosophila. Cell.

[63] Kohno H, Suenami S, Takeuchi H, Sasaki T, Kubo T (2016). Production of knockout mutants by CRISPR/Cas9 in the european honeybee, Apis mellifera l. Zool Sci.

[64] Scholl C, Kübert N, Muenz TS, Rössler W (2015). CaMKII knockdown affects both early and late phases of olfactory long-term memory in the honeybee. J Exper Biol.

[65] Hoffmann S, Otto C, Doose G, Tanzer A, Langenberger D, Christ S, Kunz M, Holdt LM, Teupser D, Hackermüller J, Stadler PF (2014). A multi-split mapping algorithm for circular RNA, splicing, trans-splicing and fusion detection. Genome Biol.

[66] Li H, Durbin R (2009). Fast and accurate short read alignment with Burrows-Wheeler transform. Bioinformatics.

[67] Gao Y, Zhang J, Zhao F. Circular RNA identification based on multiple seed matching. Brief Bioinform. 2017. 10.1093/bib/bbx014. 10.1093/bib/bbx014 28334140

[68] Reim T, Thamm M, Rolke D, Blenau W, Scheiner R (2013). Suitability of three common reference genes for quantitative real-time PCR in honey bees. Apidologie.

[69] Zdobnov EM, Tegenfeldt F, Kuznetsov D, Waterhouse RM, Simão FA, Ioannidis P, Seppey M, Loetscher A, Kriventseva EV (2016). OrthoDB v9. 1: cataloging evolutionary and functional annotations for animal, fungal, plant, archaeal, bacterial and viral orthologs. Nucleic Acids Research.

[70] Krueger F, Andrews SR (2011). Bismark: a flexible aligner and methylation caller for Bisulfite-Seq applications. Bioinforma (Oxford, England).

[71] Langmead B, Salzberg SL (2012). Fast gapped-read alignment with Bowtie 2. Nat Methods.

[72] Kozomara A, Griffiths-Jones S (2014). miRBase: annotating high confidence microRNAs using deep sequencing data. Nucleic Acids Res.

[73] Lewis BP, Burge CB, Bartel DP (2005). Conserved seed pairing, often flanked by adenosines, indicates that thousands of human genes are microRNA targets. Cell.

[74] Elsik CG, Tayal A, Diesh CM, Unni DR, Emery ML, Nguyen HN, Hagen DE (2016). Hymenoptera Genome Database: integrating genome annotations in HymenopteraMine. Nucleic Acids Res.

