# Supplementary material for: Sequence and structural properties of circular RNAs in the brain of nurse and forager honeybees (Apis mellifera)
Source: BMC Genomics. 2019 Jan 25;20:88. doi: 10.1186/s12864-018-5402-6 (PMC6347836; doi:10.1186/s12864-018-5402-6)

Additional File 1 — Splicing

Supplemental Figure 1: Intronic splice site motif. (A) Flanking introns of circRNA junctions exhibit a fairly common `ttxcAG/GTaagt` motif (visualized with WebLogo 3.4). (B) Among our high confidence circRNAs, only five showed non-canonical `AG/GT` junctions which, however, correlated with annotated exon boundaries. Acceptor sequences (yellow) are canonical while the donor sequence (gray) deviates in these cases. Small letters indicate regions marked for low complexity.

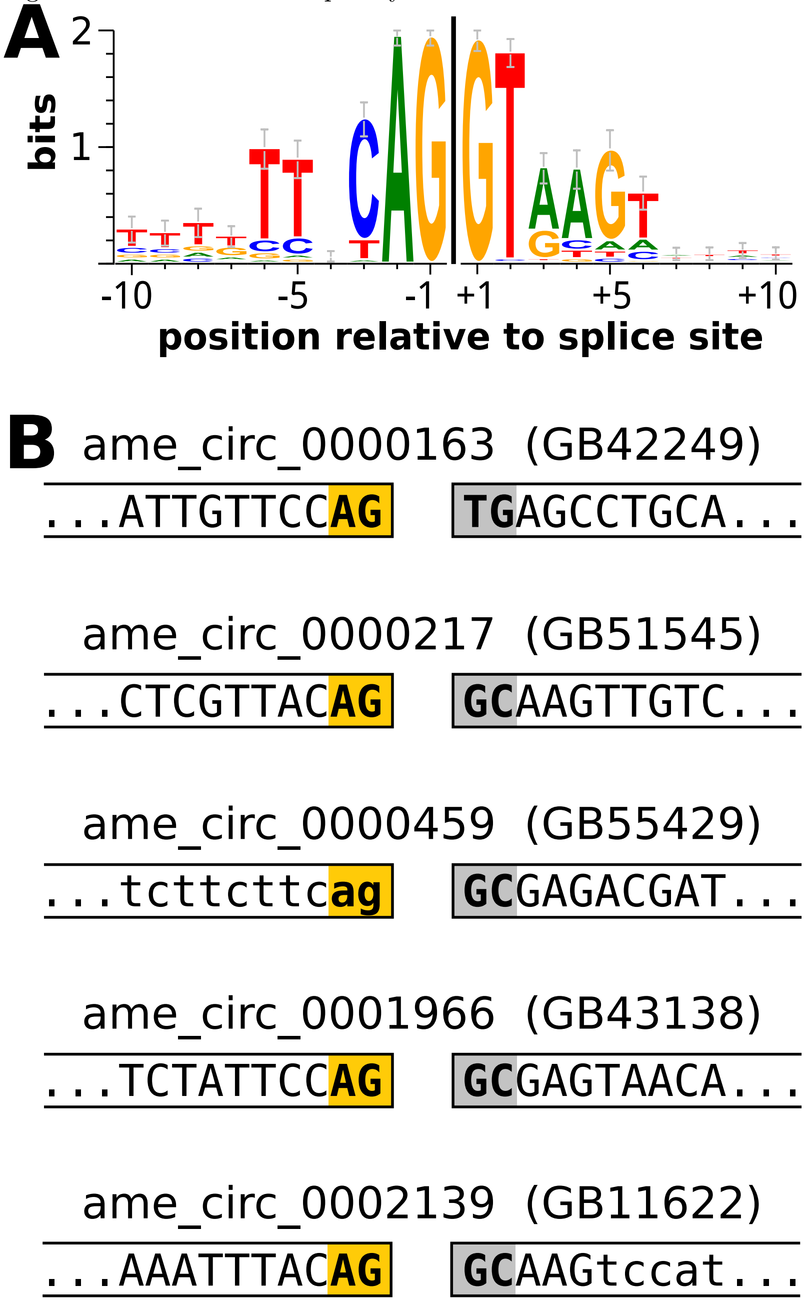

Supplemental Figure 2: Ambiguous transcript assignment. There is currently no annotated isoform of LOC408309 which includes both exons of circRNA *ame_circ_0001877*. Both exons are part of a different 5'-UTR and end with a coding region. The circRNA is well supported with 121 JSRs and likely a result of an excision from one of the longer isoforms of the transcript that contain neither of the two exons (e.g. XM_006566436.2).

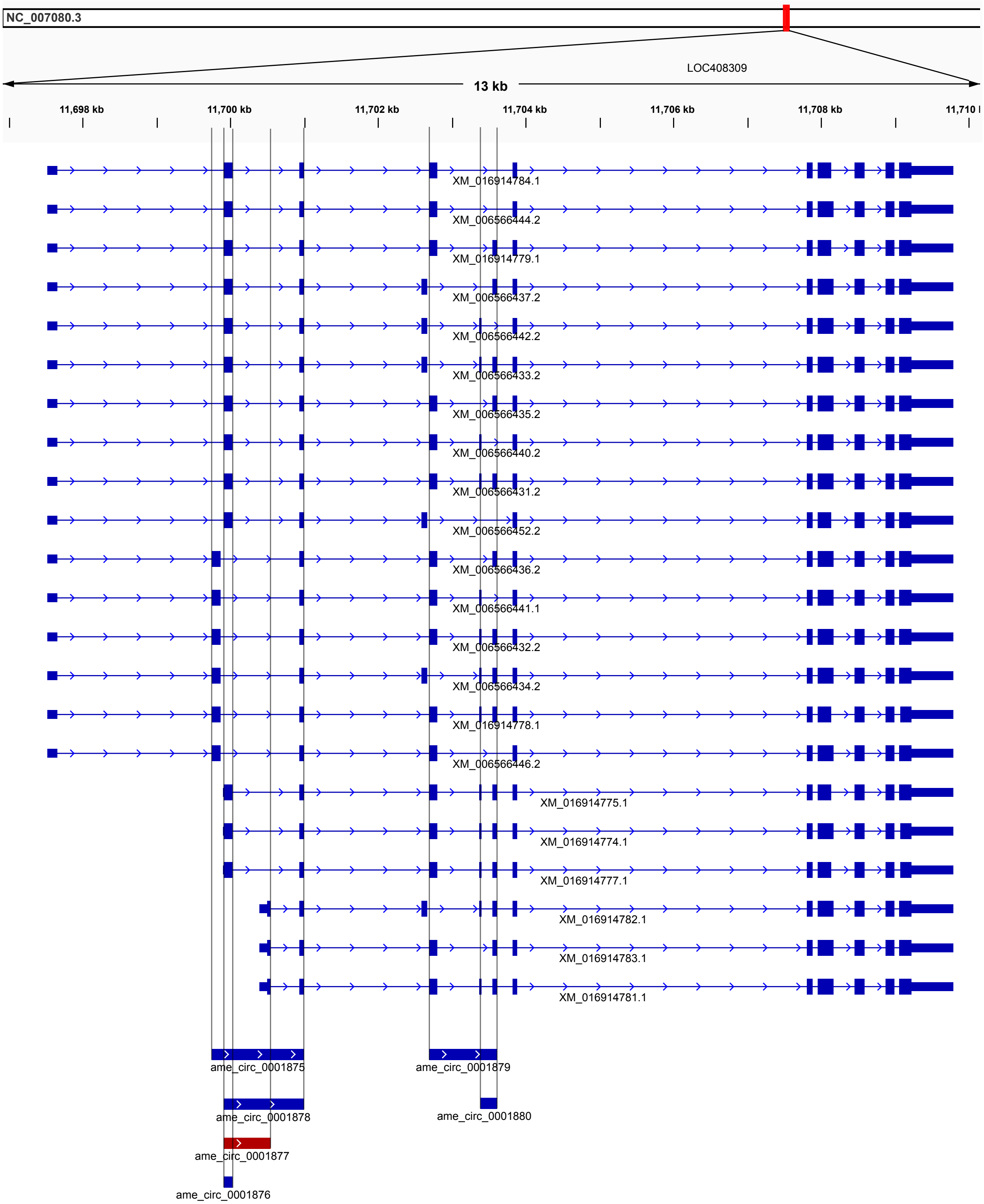

Supplement: Supplementary file 1 — Splicing. PDF showing the splice site motif of circRNAs and details on exceptions. (PDF 547 kb) [file 12864_2018_5402_MOESM1_ESM.pdf]
