# Supplementary material for: Sequence and structural properties of circular RNAs in the brain of nurse and forager honeybees (Apis mellifera)
Source: BMC Genomics. 2019 Jan 25;20:88. doi: 10.1186/s12864-018-5402-6 (PMC6347836; doi:10.1186/s12864-018-5402-6)

Additional File 6 — Verification of circRNAs via PCR

Supplemental Figure 3: Verification of circRNAs via PCR. Primer pairs amplifying a linear portion within circRNA exon (L) and a pair spanning the back-splicing junction (C) were designed for a selected number of BSJs. Anticipated PCR product length is indicated below the gels. Note that the PCR products run slightly higher than indicated by the leader because we prestained with GelRed. PCRs were carried with and without reverse transcriptase (RT) during cDNA synthesis to rule out genomic amplicons. For the five bottom BSJs RT(-) control and linear amplicons are not shown.

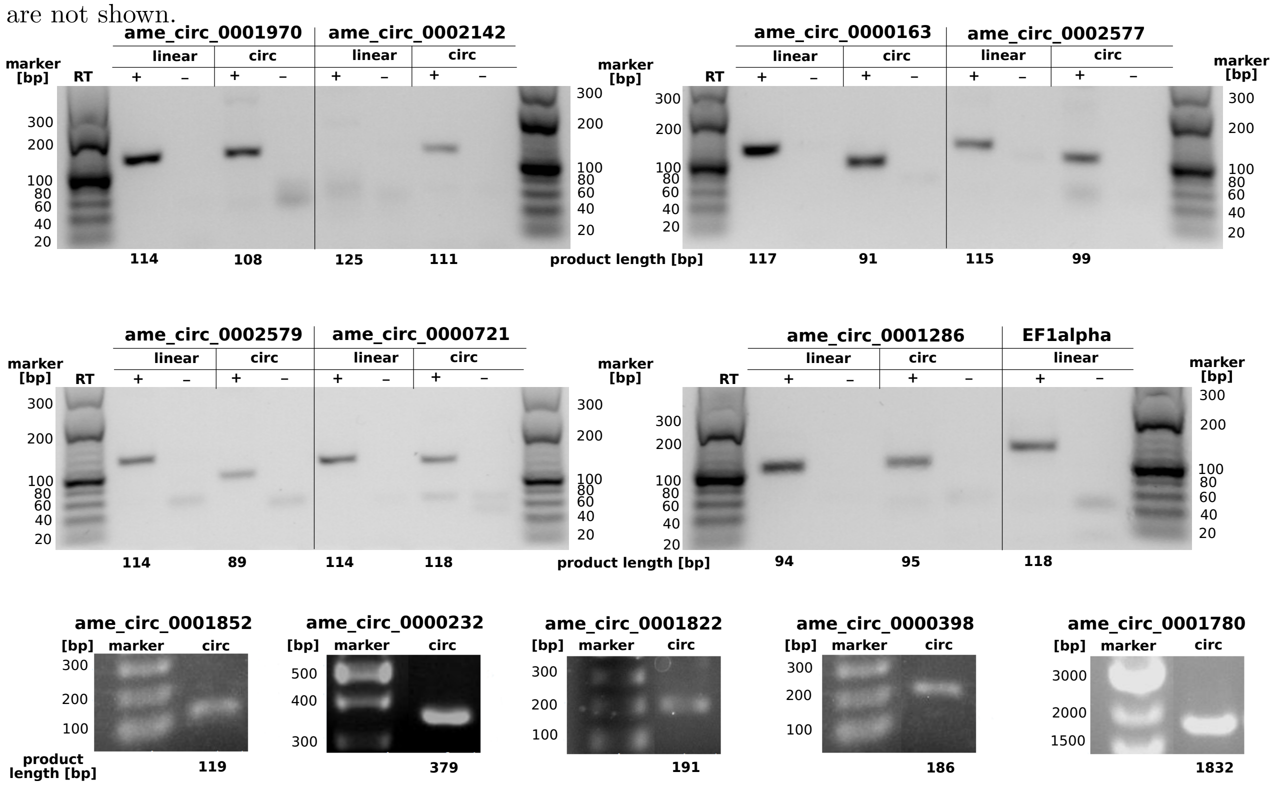

Supplement: Supplementary file 2 — Verification of circRNAs via PCR. Results of the circularity validation through PCR. (PDF 582 kb) [file 12864_2018_5402_MOESM2_ESM.pdf]
