# Supplementary material for: Sequence and structural properties of circular RNAs in the brain of nurse and forager honeybees (Apis mellifera)
Source: BMC Genomics. 2019 Jan 25;20:88. doi: 10.1186/s12864-018-5402-6 (PMC6347836; doi:10.1186/s12864-018-5402-6)

Additional File 7 — RNase R enrichment control

Supplemental Figure 3: qPCR control of circRNA enrichment. The enrichment of circRNA candidates circAm*rsmep2* and circAm*rad* using RNase R is compared to the effect on the linear circAm*ef* α. Left: The RNA concentration of the linear reference gene circAm*ef* α decreases upon RNase R treatment compared to the untreated control as indicated by increased Ct values. Reversely, the circular RNA candidate circAm*rsmep2* is efficiently enriched after RNase R treatment. Right: Similar to left but compared to circular RNA candidate circAm*rad*. Each run was performed in five technical replicates.

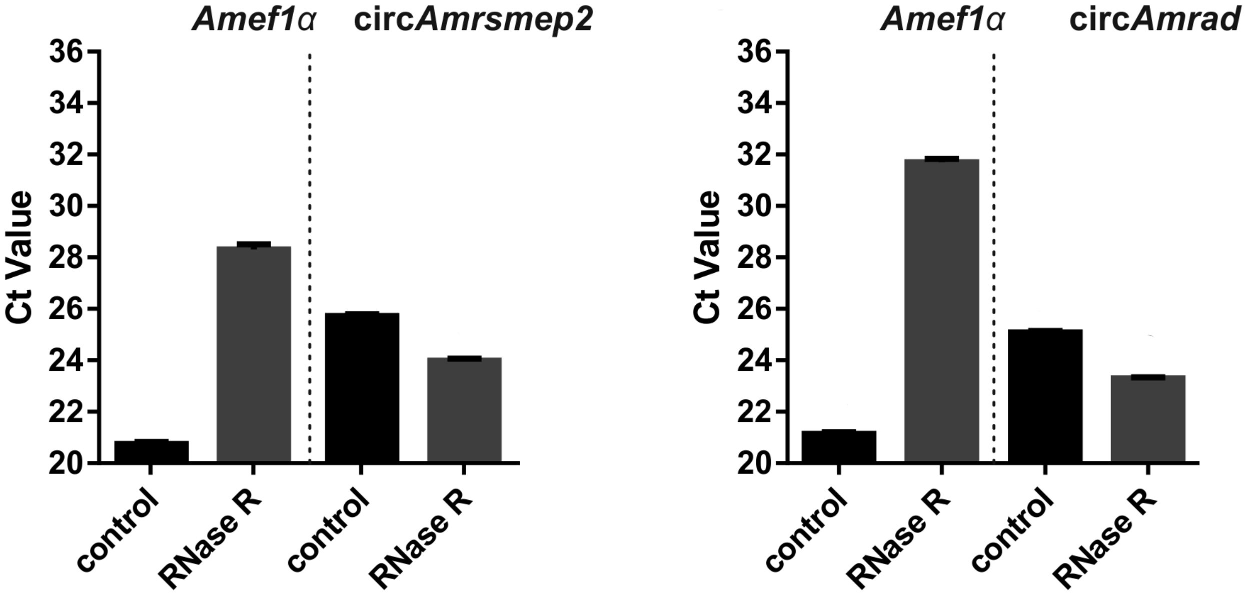

Supplement: Supplementary file 6 — RNase R enrichment control. Experimental control of circRNA enrichment over linear products. (PDF 86 kb) [file 12864_2018_5402_MOESM6_ESM.pdf]
